# The Role of the Gut Microbiome on Skeletal Muscle Mass and Physical Function: 2019 Update

**DOI:** 10.3389/fphys.2019.01435

**Published:** 2019-11-26

**Authors:** Michael S. Lustgarten

**Affiliations:** Nutrition, Exercise Physiology, and Sarcopenia Laboratory, Jean Mayer United States Department of Agriculture Human Nutrition Research Center (HNRCA), Tufts University, Boston, MA, United States

**Keywords:** muscle strength, endurance capacity, microbiome, skeletal muscle, short chain fatty acid, gut-muscle axis

## Abstract

Within the past year, several studies have reported a positive role for the gut microbiome on the maintenance of skeletal muscle mass, evidence that contrasts previous reports of a negative role for the gut microbiome on the maintenance of whole body lean mass. The purpose of this mini-review is to clarify these seemingly discordant findings, and to review recently published studies that further elucidate the gut-muscle axis.

## Role of the Gut Microbiome on Whole Body Lean Mass

A role for the gut microbiome on the maintenance of whole body lean mass, skeletal muscle mass, and physical functioning (defined as the gut-muscle axis) has been proposed by several independent research groups (de Sire et al., 2018; Grosicki et al., 2018; Ni Lochlainn et al., 2018; Picca et al., 2018; Ticinesi et al., 2019). A negative role for the gut microbiome on the maintenance of whole body lean mass was first suggested based on a pioneering study by (Backhed et al., 2004). Whole body lean mass was decreased by 7 and 9% in young male and female germ-free mice (GFM), respectively, following colonization with fecal samples from age-matched, conventionally-raised mice (Backhed et al., 2004). In support of this finding, whole body lean mass was increased following antibiotic treatment in young mice (Nobel et al., 2015). However, the impact of these interventions on the quantity of skeletal muscle mass was not reported. Whole body lean mass is commonly assumed to include muscle mass but also includes the masses of bodily tissues, including the heart, liver, kidneys, and intestine. Interestingly, cecum hypertrophy has been reported following antibiotic treatment in mice, an intervention that reduces the colonic expression of antimicrobial factors to levels found in germ-free mice (Reikvam et al., 2011). More specifically, cecum weight was 5.6-fold increased (1,804 vs. 325 mg) in young antibiotic-treated mice, an effect that was reversed following natural microbiota reseeding (removal of antibiotic treatment in conjunction with exposure of these mice to soiled litter from control mice) (Nay et al., 2019). Based on these findings, whether skeletal muscle mass was increased in the studies of Backhed and Nobel (Backhed et al., 2004; Nobel et al., 2015) is unknown because the higher levels of whole body lean mass in GFM and in antibiotic-treated mice may simply be due to an enlarged cecum.

## Role of the Gut Microbiome and Short-Chain Fatty Acids on Skeletal Muscle Mass

In contrast, several studies have been published within the past year that demonstrate a positive role for the gut microbiome on the maintenance of skeletal muscle mass. In support of this, the muscle mass/body weight ratio was reduced in young GFM, and colonization of GFM with fecal samples from age-matched conventionally raised mice restored the muscle mass/body weight ratio (Lahiri et al., 2019). Similarly, in antibiotic-treated young mice, muscle mass was reduced (Manickam et al., 2018; Nay et al., 2019; Okamoto et al., 2019) without a corresponding change in body weight (Manickam et al., 2018; Okamoto et al., 2019), thereby resulting in a decreased muscle mass/body weight ratio (Nay et al., 2019). In addition, muscle mass and the muscle mass/body weight ratio were increased following natural microbiota seeding in antibiotic-treated mice (Nay et al., 2019). In terms of bacterial species that may positively impact muscle mass, oral gavage with *Lactobacillus casei* or *Bifidobacterium longum* increased the muscle mass/body weight ratio without affecting body weight (Ni et al., 2019).

Which bacterial factors may positively affect the maintenance of skeletal muscle mass? The muscle mass/body weight ratio was increased in young GFM fed a mixture of the SCFAs, acetate, propionate, and butyrate, when compared with control-fed GFM (Lahiri et al., 2019). Propionate and butyrate are found in the colonic lumen of conventionally raised, but not germ-free mice (Matsumoto et al., 2012), evidence that identifies them as bacterially-derived metabolites. Similarly, muscle mass was increased without a corresponding change in body weight, thereby increasing the muscle mass/body weight ratio in aged mice (26 months old) that were fed butyrate for 10 months, when compared with unsupplemented controls (Walsh et al., 2015).

## Role of the Gut Microbiome and Short-Chain Fatty Acids on Physical Function

A role for the gut microbiome on physical functioning, including muscle strength and endurance exercise capacity has been reported in seven studies within the past year. Grip strength was decreased in young GFM, when compared with age-matched, conventionally-raised mice (Lahiri et al., 2019). Treadmill endurance capacity was reduced in conjunction with increased *ex vivo* muscle fatigability in antibiotic-treated mice (Nay et al., 2019; Okamoto et al., 2019), and swimming endurance capacity was reduced in young GFM, when compared with conventionally-raised mice (Huang et al., 2019). In terms of bacterial taxa that may underlie the maintenance of physical function, oral gavage with the bacterial species *Lactobacillus casei* or *Bifidobacterium longum* increased grip strength in young mice (Ni et al., 2019). Colonization of young GFM with *Eubacterium rectale* or *Clostridium coccoides* increased swim time to exhaustion, when compared with uncolonized GFM (Huang et al., 2019). An increase in the bacterial genus *Veillonella* was observed in human marathon runners post-marathon, and colonization of mice with the bacterial species *Veillonella atypica* increased treadmill run time to exhaustion (Scheiman et al., 2019). As a potential mechanism for how *V. atypica* may improve endurance exercise capacity, intra-rectal instillation of the SCFA propionate similarly increased treadmill run time (Scheiman et al., 2019). Separately, acetate infusion in antibiotic-treated mice improved treadmill endurance capacity (Okamoto et al., 2019). Moreover, grip strength was increased in GFM fed a SCFA mixture, when compared with conventionally-raised, control-fed mice (Lahiri et al., 2019). However, whether SCFAs can affect physical function in aged animals is less clear. Butyrate supplementation was not able to reverse the age-related decrease in grip strength found in aged mice (Walsh et al., 2015).

It is important to note that with the exception of (Walsh et al., 2015), the studies referenced in this mini-review have been performed in young mice and humans. Studies aimed at investigation of the gut-muscle axis in older adults are limited, as discussed in previous reviews (de Sire et al., 2018; Grosicki et al., 2018; Ni Lochlainn et al., 2018; Picca et al., 2018; Ticinesi et al., 2019). Recent findings from our group add to elucidation of the gut-muscle axis in older adults. We identified higher levels of *Prevotellaceae*, *Prevotella*, *Barnesiella*, and *Barnesiella intestinihominis* in older adults in conjunction with higher muscle strength (defined as high-functioning, HF), when compared with older adults that had reduced muscle strength (defined as low-functioning, LF) (Fielding et al., 2019). Moreover, to evaluate a causative role for the gut microbiome on muscle strength, we transplanted fecal samples from HF and LF older adults into GFM, and similar differences for these bacteria were identified when comparing their respectively colonized mice, in conjunction with higher muscle strength in HF-colonized mice. Interestingly, *Barnesiella* and *Prevotellaceae* contain genes that produce acetate, propionate, and butyrate (Morotomi et al., 2008; Chen et al., 2017; Esquivel-Elizondo et al., 2017; Louis and Flint, 2017). However, whether SCFAs positively affect muscle strength in older adult humans is unknown.

## Discussion

Collectively, these studies suggest that increasing gut bacterial SCFA production may positively affect skeletal muscle mass and physical function in humans. Two approaches for increasing gut bacterial SCFA production include a high-fiber diet and exercise. First, acetate, propionate, and butyrate production are increased following fiber fermentation by gut bacteria (Bourquin et al., 1993). Interestingly, when compared with consumption of a high-fiber diet, a low-fiber diet reduced muscle mass without altering body weight and decreased treadmill endurance capacity in young mice in conjunction with decreased *Prevotellaceae*, *Prevotella*, and fecal SCFAs (Okamoto et al., 2019). Although dietary fiber intake is positively associated with handgrip strength and physical functioning in older adult humans (Wu et al., 2013; Tak et al., 2018), few studies aimed at increasing dietary fiber intake, quantifying fecal SCFAs, and examining the resultant effects on skeletal muscle mass or physical function have been reported. To date, only one study has explored this hypothesis: older adults that consumed the fermentable fiber, inulin had increased grip strength (Buigues et al., 2016), but fecal SCFAs were not quantified. Second, fecal SCFAs were increased in response to 6 weeks of aerobic exercise training in young adult humans in conjunction with improvements in body composition and physical functioning (Allen et al., 2018), but few related studies in older adults have been published. Of note, *Bacteroides* were increased in older adults in response to 12 weeks of endurance exercise training (which improved cardiorespiratory fitness) (Morita et al., 2019), but fecal SCFAs were not measured.

In sum, with the goal of further elucidating the gut-muscle axis in older adult humans, future studies aimed at increasing fecal SCFA production (whether through dietary fiber, exercise, or both) and evaluating the impact on muscle mass and physical function are of interest.

## Author Contributions

The author confirms being the sole contributor of this work and has approved it for publication.

### Conflict of Interest

The author declares that the research was conducted in the absence of any commercial or financial relationships that could be construed as a potential conflict of interest.
